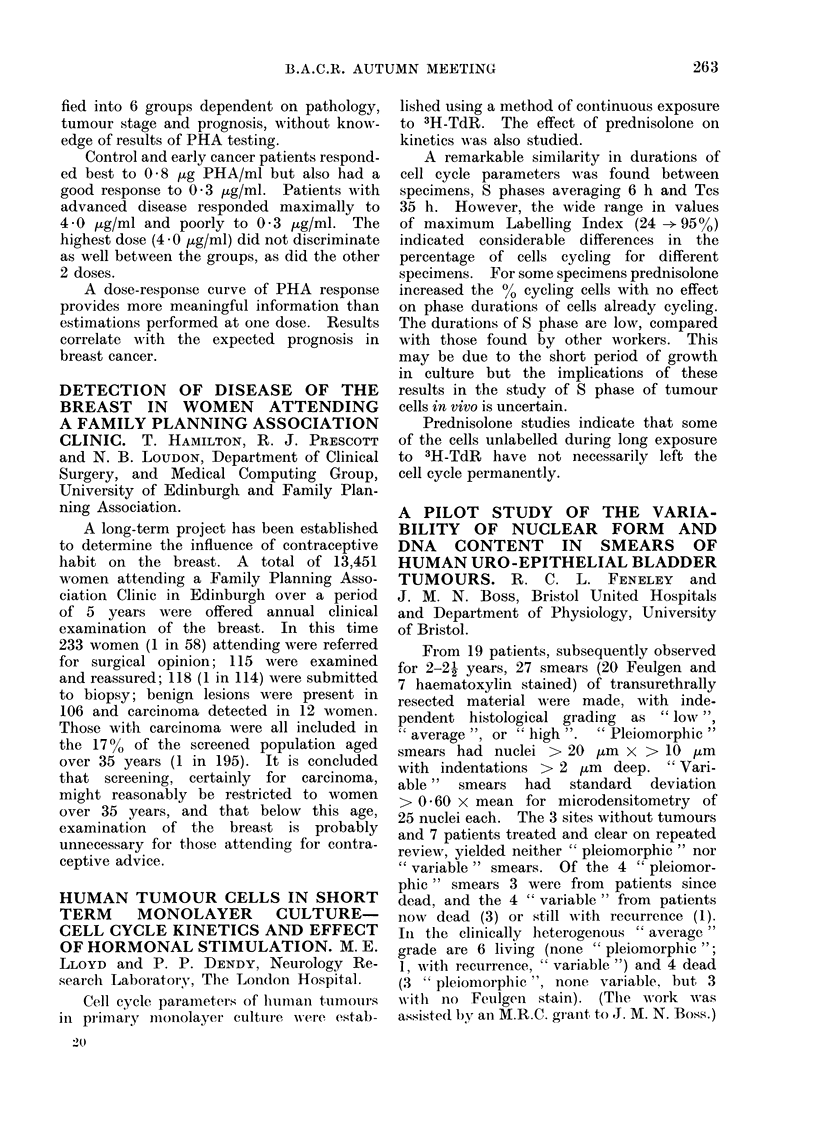# Proceedings: Human tumour cells in short term monolayer culture-cell cycle kinetics and effect of hormonal stimulation.

**DOI:** 10.1038/bjc.1975.47

**Published:** 1975-02

**Authors:** M. E. Lloyd, P. P. Dendy


**HUMAN TUMOUR CELLS IN SHORT TERM MONOLAYER CULTURE—CELL CYCLE KINETICS AND EFFECT OF HORMONAL STIMULATION.** M. E. LLOYD and P. P. DENDY, Neurology Research Laboratory, The London Hospital.

Cell cycle parameters of human tumours in primary monolayer culture were established using a method of continuous exposure to $^3$H-TdR. The effect of prednisolone on kinetics was also studied.

A remarkable similarity in durations of cell cycle parameters was found between specimens, S phases averaging 6 h and Tcs 35 h. However, the wide range in values of maximum Labelling Index (24 → 95%) indicated considerable differences in the percentage of cells cycling for different specimens. For some specimens prednisolone increased the % cycling cells with no effect on phase durations of cells already cycling. The durations of S phase are low, compared with those found by other workers. This may be due to the short period of growth in culture but the implications of these results in the study of S phase of tumour cells *in vivo* is uncertain.

Prednisolone studies indicate that some of the cells unlabelled during long exposure to $^3$H-TdR have not necessarily left the cell cycle permanently.